# Future Path Presentation to Passengers of an Autonomous Wheelchair Using Vibrotactile Feedback

**DOI:** 10.3390/s25061714

**Published:** 2025-03-10

**Authors:** Yusuke Higashi, Hiroyuki Takai, Tetsushi Ikeda

**Affiliations:** Graduate School of Information Sciences, Hiroshima City University, Hiroshima 731-3194, Japan; higashi@sr.info.hiroshima-cu.ac.jp (Y.H.); takai@hiroshima-cu.ac.jp (H.T.)

**Keywords:** autonomous wheelchairs, vibrotactile feedback, future path notification, path visualization, passenger comfort and safety, tactile apparent motion

## Abstract

While autonomous wheelchairs reduce the burden on passengers, automation can make it difficult for them to anticipate the future path of the wheelchair, potentially causing anxiety or discomfort due to unexpected movements. In this study, we define “path” as the geometric trajectory of the wheelchair position, without considering temporal aspects. Providing passengers with information about this future path is crucial, particularly when multiple pedestrians or obstacles are present. Previous studies have primarily focused on presenting only the direction in which the wheelchair turns. In this study, we propose a path presentation method that conveys both the direction and width of turns by varying the duration of haptic apparent motion according to the turning width. The results from the evaluation experiment showed that presenting the future path, including the extent of avoidance maneuvers, improved user understanding and offered a slightly greater sense of security compared to methods that presented only directional information or no feedback at all.

## 1. Introduction

The development of autonomous wheelchairs has been extensively studied to broaden the range of activities and improve the quality of life for individuals with mobility impairments [1,2,3]. Compared to manually operated electric wheelchairs, autonomous systems offer several advantages, such as reducing the burden on users who may feel anxious about driving, minimizing accidents caused by human error, and enabling safer mobility. These benefits position autonomous wheelchairs as a promising transportation solution for elderly individuals and those with physical disabilities.

However, a significant challenge lies in the reduced involvement of passengers in the driving process, which can make the behavior of the wheelchair appear unpredictable and unsettling. Users of manually operated wheelchairs can anticipate and control the movements of their device, whereas passengers of autonomous wheelchairs often face uncertainty regarding the decision-making of the system. For instance, they may be unaware of the specific path the system will choose to avoid pedestrians or obstacles in the environment. In this study, “path” refers to the geometric trajectory of the position of the wheelchair, independent of time.

To address this issue, presenting the planned future behavior of the wheelchair to passengers can be effective. Numerous studies have investigated methods for presenting information to drivers of automated vehicles [4,5,6]. Unlike cars, wheelchairs primarily travel on sidewalks and in public spaces, encountering various paths to avoid pedestrians and obstacles. Therefore, it is crucial to present path planning information to users of autonomous wheelchairs, including the timing and width of avoidance maneuvers.

This study aims to propose a method for presenting future behavior to passengers of an autonomous wheelchair using vibrotactile feedback and to verify its effectiveness through implementation. The proposed method employs an array of vibration devices installed on the back of the wheelchair seat to convey information about the behavior of the wheelchair. A distinctive feature of this approach is its reliance on vibrotactile feedback, which avoids interference with the user’s perception of the surrounding environment, unlike audio-visual presentations. Unlike conventional systems that merely indicate the direction of the turns of the wheelchair, this method also provides detailed path information, including the width of the path to be avoided. This approach is designed to help passengers understand both the timing of planned avoidance maneuvers and the intended path of the wheelchair, enhancing their awareness and confidence during travel. This paper extends the method proposed in [7] and validates it in a new experiment using a wheelchair robot.

The contributions of this paper are summarized as follows:

1. We propose a method for presenting a future travel path to passengers of an autonomous wheelchair using an array of tactile devices installed onff the seat.
2. We evaluate the proposed method in scenarios where the wheelchair automatically moves left or right to avoid pedestrians and an obstacle.
3. We demonstrate that the proposed method enables passengers to more easily understand the future action plan compared to conventional methods, which only indicate the direction of movement.

The remainder of this paper is organized as follows: Section 2 reviews related work, Section 3 describes the proposed method, Section 4 and Section 5 detail the validation experiment and report the results, respectively. Section 6 presents a discussion of the results, and finally, Section 7 concludes the paper.

## 2. Related Work

### 2.1. Presentation of Vehicle Behavior to Passengers in Automated Driving

The importance of evaluating passenger comfort in automated driving, particularly for individuals not involved in driving operations, has been widely recognized. Elbanhawi et al. [8] highlighted the need to redesign frameworks for assessing passenger comfort as the loss of control experienced by passengers in automated vehicles can significantly affect their overall comfort. Studies have also examined the conditions under which passenger comfort decreases during automated driving and have explored interface design solutions to mitigate these effects [4,9,10]. When drivers are not actively engaged in vehicle operation, their perception of the surroundings of the vehicle tends to diminish [11], potentially leading to anxiety and discomfort due to an inability to predict the behavior of the vehicle.

To address this problem, it is essential to share the planned future behavior of the driving system with passengers of automated wheelchairs [5]. Hashimoto et al. [12] investigated information presentation methods for passengers of automated wheelchairs and demonstrated that displaying the future behavior of the wheelchair reduced stress levels, as measured by physiological indices. Watanabe et al. [13] proposed presenting future travel paths to passengers via ground projections, which increased the sense of security for both passengers and surrounding pedestrians [14]. However, projection-based presentations face challenges in crowded environments, where projections are often obstructed by pedestrians. Yoshitake et al. [15] demonstrated that presenting a future 3 s path could reduce user anxiety, but this method was only validated in simulations and not implemented in an actual wheelchair system. Additionally, conventional information presentation methods rely primarily on visual or auditory channels [9]. Visual displays may draw passengers’ attention away from their surroundings due to the need to focus on the display. Shiomi et al. [16] used voice guidance for the automated operation of electric wheelchairs to assist users in navigating complex paths, such as narrow passageways and elevators. Nevertheless, auditory presentations are less effective in noisy environments, where ambient noise can render them inaudible.

This study proposes and evaluates a method for presenting detailed future behavior of an automated wheelchair to its passenger using a vibration-based device. The method shares the planned path of the driving system with the passenger, including details such as when the wheelchair will avoid obstacles, which side it will move to, and the extent of the avoidance. Conventional experiments on presenting path information to passengers of automated wheelchairs have primarily focused on providing warnings, such as indicating right/left turns or stops. However, wheelchairs often operate in lane-less environments, where multiple potential paths exist in situations requiring avoidance maneuvers or speed adjustments to navigate around pedestrians or obstacles. For passengers who are not involved in the operation of the wheelchair, it is crucial to present more detailed future path information to enhance their understanding and comfort.

### 2.2. Presenting Information Through Haptic Feedback

The effectiveness of using tactile feedback to present information to car drivers has been well demonstrated [17,18]. van Erp and van Veen [19] showed that tactile path displays reduced driver workload compared to visual displays, particularly for drivers under high cognitive load. Stanley [20] demonstrated that tactile warnings for lane departures resulted in shorter reaction times than acoustic warnings. Scott et al. [21] compared rear crash warnings presented via visual, auditory, and tactile modalities, finding that tactile warnings produced the shortest average reaction times. Chang et al. [22] placed vibration devices on the seat and backrest, evaluating reaction speed and the ease of understanding information such as driving straight or turning left or right. They found that tactile feedback resulted in shorter reaction times and was subjectively easier to understand compared to visual or auditory methods. These findings indicate that tactile feedback has clear advantages in terms of both the speed of information transmission and the ease of comprehension.

Building on the advantages of tactile information presentation, numerous studies have explored its use for conveying navigation information to drivers, such as the location of surrounding vehicles and obstacles, right and left turns, and other guidance to reach a destination.

As a method for conveying path information through tactile feedback, Tan et al. [23] used a 3 × 3 vibrating array on the back of a seat to present directional movement. van Erp et al. [24] employed a tactile waist belt to provide distance and directional information for upcoming turns. Similarly, Asif et al. [25] utilized a vibrating belt, where the position of the vibrating device indicated the turning direction, and the number of vibration pulses represented the distance to the turn. In a study involving wheelchair passengers, Chen and Agrawal [26] evaluated the effectiveness of force feedback applied to a joystick as a navigation aid. Devigne et al. [27] proposed a method for presenting obstacle directions and safe travel paths during power wheelchair operation. Conventional tactile information systems have thus primarily focused on providing navigation information to drivers, such as the location of surrounding vehicles and obstacles or the direction of right and left turns, for manually operated vehicles.

In contrast, an automated wheelchair operates in a lane-less environment, making it crucial to present not only information about right or left turns but also details that enable passengers to predict the future path of the wheelchair. However, conventional studies on tactile information presentation for automated wheelchairs have primarily focused on providing passengers with information about upcoming turns and stops, without verifying the effectiveness of presenting detailed future travel paths.

This study proposes and experimentally evaluates a method for presenting future path information to passengers of automated wheelchairs using an array of vibrotactile sensations. This method conveys an intuitive representation of the planned path of the wheelchair, including maneuvers to avoid pedestrians and obstacles. We proposed a method based on this approach in [7], but some participants had difficulty perceiving the vibrations, and challenges remained in installing the vibration device. Additionally, a presentation method for various avoidance widths was not developed. Furthermore, in the evaluation experiment in [7], only a single fixed obstacle was considered, pedestrian avoidance was not evaluated, and no comparison was made with a condition without vibration presentation. In this paper, we propose an improved installation method that enhances vibration transmission by optimizing the device arrangement. We also introduce a method for presenting various avoidance widths, conduct an evaluation experiment where multiple targets, including pedestrians, are avoided, and compare the results with a condition without vibration presentation.

## 3. Future Path Presentation Using Vibrotactile Sensations

In this section, we propose a method for presenting a future travel path to passengers using a vibration device installed on the backrest of an autonomous wheelchair. We describe how an array of vibrating devices can be utilized to indicate both the timing and manner of avoiding pedestrians and obstacles during travel.

### 3.1. Information Presentation for Autonomous Wheelchair Passengers

**Figure 1:**
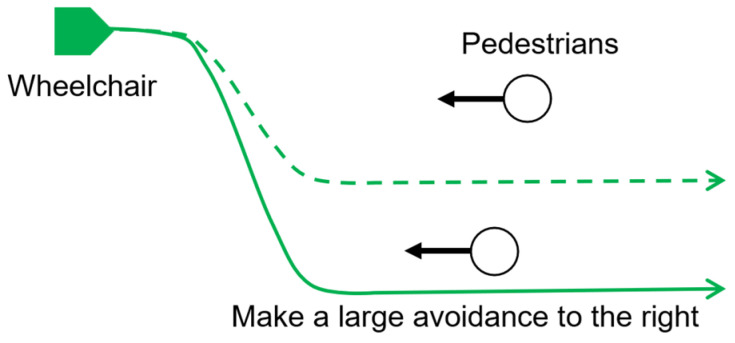
Example of multiple potential paths when the wheelchair avoids pedestrians. Possible paths include a large detour (solid line) and a path passing between pedestrians (dotted line). Passengers may feel anxious if the path is unknown.

Figure 1 illustrates a scenario in which an autonomous wheelchair is navigating its environment. In this situation, there are multiple possible paths that the wheelchair could take to avoid moving to the right. Simply providing information about rightward avoidance is insufficient. As shown in the figure, two pedestrians approach from the right, and the left-facing wheelchair avoids the pedestrian in front of it by moving to the right. The wheelchair may either take a slight detour to avoid the first pedestrian (dotted path) or move further to the right to avoid the second pedestrian (solid path).

Providing passengers with advance information about the intended path of the wheelchair can help alleviate their anxiety and discomfort. It is essential to inform passengers of the planned avoidance paths under various circumstances to enhance their sense of security and understanding.

### 3.2. Presenting Future Behavior via Vibration Propagation

**Figure 2:**
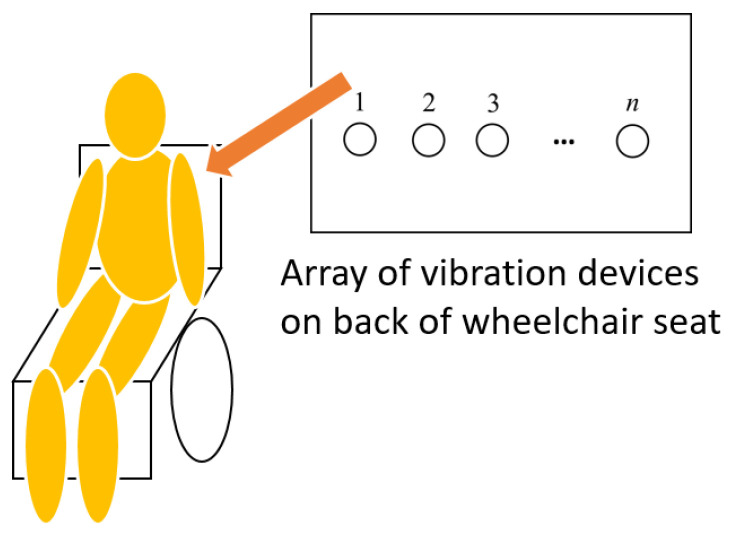
Arrangement of an array of vibration devices on the backrest of the wheelchair seat to present future travel paths using vibrations.

This study proposes a method for presenting detailed future travel paths of a wheelchair using an array of vibration devices installed on the seat. Figure 2 illustrates the arrangement of these devices, which are placed horizontally. Travel paths are conveyed by sequentially activating the vibrating devices in the array. Specifically, the direction of sequential vibrations indicates the direction in which the wheelchair avoids obstacles, while the duration of the vibrations represents the degree of avoidance. This approach allows passengers to visualize the planned avoidance path as the wheelchair maneuvers left or right to avoid collisions with obstacles or pedestrians.

**Figure 3:**
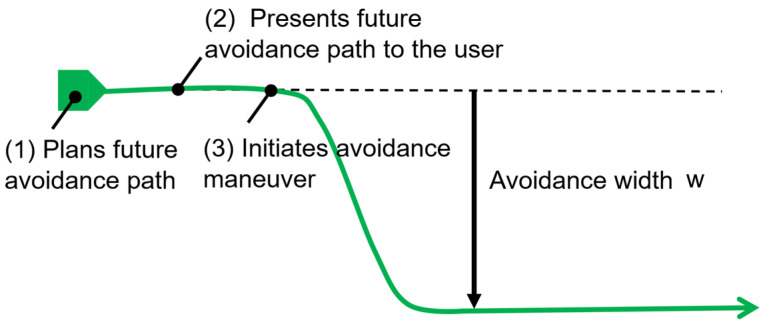
Presentation of the avoidance width of the wheelchair based on delay time.

Figure 3 illustrates the process of planning an avoidance path to the right as the wheelchair moves from left to right. The green line in the figure represents the planned path. In the proposed method, once the wheelchair determines the future avoidance path (Figure 3(1)), it presents the direction and width of the avoidance to the passenger (2), and subsequently, the wheelchair follows the planned path by changing the actual trajectory (3).

**Figure 4:**
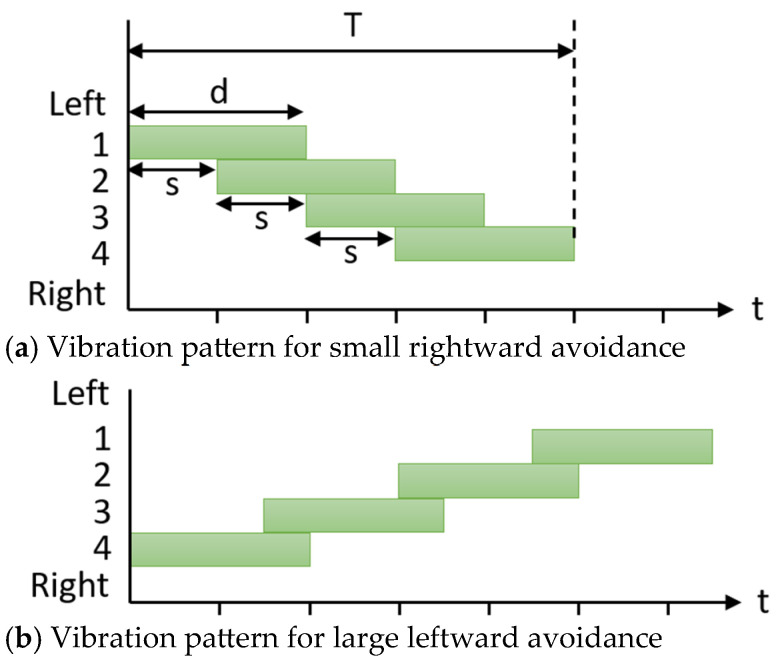
Vibration patterns presented by the proposed method to indicate the avoidance width of the wheelchair.

Figure 4a shows an example of a vibration pattern that communicates an avoidance path to the right using an array of four vibration devices. The horizontal axis represents time, while the vertical axis represents the vibrating device number. This example illustrates how the sequential activation of vibrating devices from left to right indicates that the wheelchair will avoid turning to the right. The total vibration duration conveys the width of the avoidance path. The total vibration duration is measured from the activation of the first vibration device to the deactivation of the last device. The total vibration time *T* is determined by the duration of the activation of each vibration device (vibration time *d*) and the interval between the activation of successive devices (delay time *s*).

In the proposed method, the vibration time *d* is constant, while the delay time *s* and the total vibration time *T* are determined by the following equations:

(1) s=αw+β

(2) T=d+(n−1) s 

where *w* represents the avoidance width shown in Figure 3, α and β are constants that define the delay time as a linear function, and *n* is the number of vibrating devices arranged in a row. Assume that *w* takes values in the range where *s* does not exceed *d*. In the experiments described in the next section, the number of vibrating devices is *n* = 4, with constants α=0.3 and β=−0.15 used to determine the delay time. If *s* < 0, the avoidance width of the wheelchair is minimal and is not presented to the user.

The example in Figure 4a illustrates the information presented for a small avoidance to the right. The total vibration time is short, indicating a minor rightward avoidance. Figure 4b shows an example of a larger leftward avoidance compared to Figure 4a. To ensure the passenger clearly understands the intended path, the vibration pattern shown in Figure 4 is repeated twice before the wheelchair reaches the position indicated in Figure 3(3), where the actual avoidance maneuver is executed.

The proposed method does not aim to convey the position of individual vibrating devices but instead focuses on the smooth presentation of continuous vibration movement.

To achieve this, the method leverages the concept of apparent motion, an illusion of motion created by the discrete stimulation of points appropriately spaced in time and location [28]. In tactile perception, apparent motion occurs when a stimulus is perceived as moving continuously from one point to another if two distinct points on the skin are stimulated with a time delay. The conditions for apparent motion are determined by two factors: the duration of vibration for each device (vibration time *d*) and the time interval between the onset of vibration at the preceding stimulus point and the following stimulus point (delay time *s*). In this study, the vibration time *d* is fixed, while the delay time *s* is varied within the range *s* < *d*. This variation is intended to create a perception movement along the path. The objective of this approach is not to ensure the continuous perception of motion, but rather to present a pathway that can be clearly interpreted by the participant.

### 3.3. Timing of Future Path Presentation

The timing of presenting avoidance path information through vibratory motion is critical to ensuring passenger understanding and comfort. Previous studies have shown that providing information 3 s before travel reduces passenger anxiety [15] and is considered an appropriate timing [14,29]. With the proposed presentation method, the total stimulus time varies depending on the situation. If the information is presented too far in advance, passengers may be unable to anticipate when the wheelchair will act, potentially increasing their anxiety.

In a previous study, we found that passengers could easily predict the behavior of the wheelchair and felt a higher sense of security when the autonomous wheelchair began turning immediately after the vibration presentation ended [7]. Based on these findings, this study presents path information at the moment the wheelchair initiates right or left turning behavior, immediately following the vibration presentation.

## 4. Experiment

To evaluate the effectiveness of the proposed future path presentation method, an experiment was conducted in a scenario where a wheelchair navigated around obstacles and pedestrians. The proposed method was compared to two conditions: one in which only the direction of avoidance was presented and another in which no information was provided, based on the passenger’s subjective evaluation.

### 4.1. Experimental Environment

**Figure 5:**
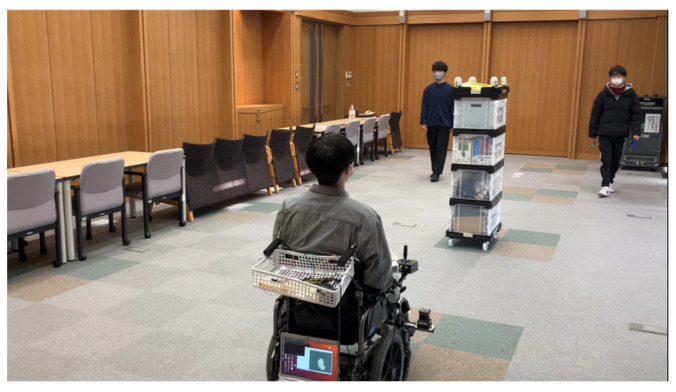
Experimental scene.

**Figure 6:**
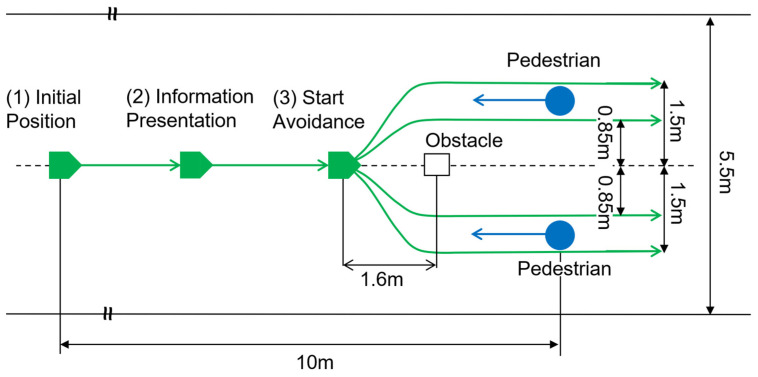
Path and layout diagram of the wheelchair. The wheelchair selects one of four paths to avoid obstacles and pedestrians.

The wheelchair operated autonomously, navigating around obstacles and pedestrians during its movement. Figure 5 shows the experimental scene, while Figure 6 provides the layout of the environment. The green lines in Figure 6 represent the paths taken by the wheelchair during the experiment. Starting from position (1) in Figure 6, the wheelchair selected a path to avoid the obstacle. At position (2), it presents information about the future path to the passenger. The wheelchair then initiates left or right movement to avoid the obstacle at position (3). Vibrotactile feedback conveying the avoidance path is delivered to the passenger, with the vibration ending just before the wheelchair reaches position (3).

The wheelchair followed one of four possible paths, including a path between the obstacle and the pedestrian or a larger detour around the pedestrian. The wheelchair maintained a constant speed except for slight deceleration when changing direction. A fixed obstacle was placed in the straight path of the wheelchair after the starting position. Two pedestrians, played by experimenters, approached the wheelchair from the front. These pedestrians walked straight from the same starting position to the left in Figure 6 at a constant speed across all trials. During the experiment, the wheelchair navigated along the four predefined paths without any contact with the pedestrians. To ensure natural pedestrian behavior, the pedestrians moved in a way that required the wheelchair to make slight avoidance maneuvers when passing.

### 4.2. Condition

The wheelchair presented three types of information to the passenger, as outlined in Table 1, and the differences between these conditions were compared. We evaluated whether the proposed method (Condition A), which included future path presentation, improved the passenger’s understanding of the path and increased their sense of safety compared to Conditions B and C. Additionally, we examined whether presenting directional information (Condition B) provided any benefit in path comprehension and safety perception compared to the absence of information (Condition C).

**Figure 7:**
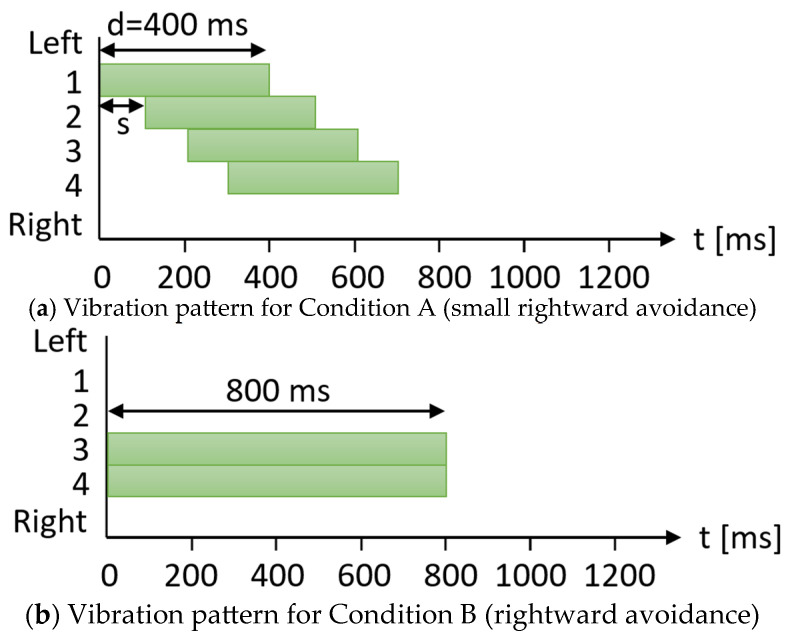
Examples of vibration patterns in experimental conditions A and B.

Figure 7 illustrates an example of the vibration pattern used to convey rightward avoidance to the passenger. The horizontal axis represents time, while the vertical axis represents the device number that vibrates. Referring to a previous study [6], we set the vibration time to *d* = 400 ms. The number of vibration devices was set to *n* = 4 based on the current setup, and the constants for determining the delay time to α=0.3 and β=−0.15, as the avoidance width was expected to range between 0.5 m and 1.8 m in the environment. The wheelchair completed two consecutive trips under each condition: one on the 1.5 m avoidance path and one on the 0.85 m avoidance path, as shown in Figure 6. These constants can be adjusted based on the environment in which the device is used. In this experimental setup, two avoidance widths of the wheelchair (0.85 m and 1.50 m) were used.

Figure 7a illustrates the presentation when the wheelchair conveys an avoidance direction and an avoidance width of 0.85 m under Condition A. The delay time *s* was set following the method described in Section 3 and was determined to be *s* = 105 ms. For an avoidance width of 1.50 m, the delay time was set to *s* = 300 ms. In Condition B, where only the left and right avoidance directions were presented, the direction of avoidance was indicated by vibrating the device to the right of the center of the seat (Figure 7b). The total vibration duration of the vibrating devices is the same in Conditions A and B. This is demonstrated by the equal total area of the rectangles in Figure 7a,b.

### 4.3. Apparatus

#### 4.3.1. Wheelchair Robot

**Figure 8:**
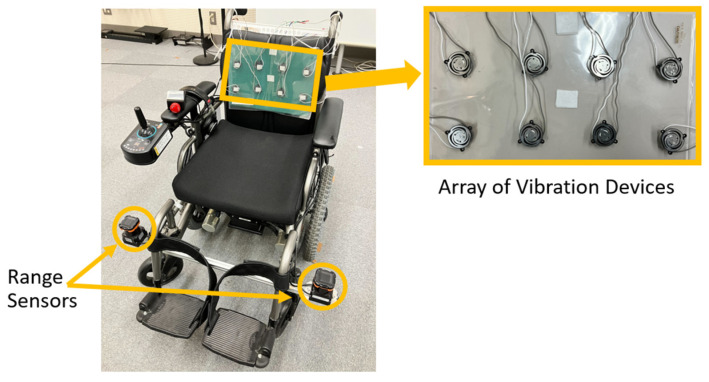
The wheelchair robot used in the experiment. Vibration devices were installed on the seat of the wheelchair.

Figure 8 shows the electric wheelchair used in the experiment, NEO-PR (Nissin Medical Industries Co., Ltd., Aichi, Japan). The wheelchair operated autonomously along a predefined path under computer control, using two UTM-30LX range sensors (HOKUYO AUTOMATIC CO., LTD., Osaka, Japan) mounted at a height of 34 cm. It localized its position and orientation by matching sensor observations with a pre-acquired grid map of the environment using a particle filter [30]. The wheelchair robot traveled at a maximum speed of 0.8 m/s during each experimental condition.

#### 4.3.2. Vibration Device Installation and Vibration Control

The vibration devices were mounted on a seat attached to the wheelchair (Figure 8). A thin sheet was placed between the vibration devices and the passenger to prevent direct contact with the passenger’s clothing, which could potentially damage the clothing or the devices.

**Figure 9:**
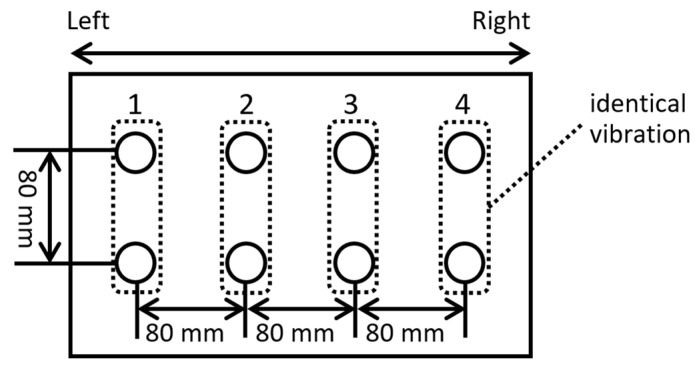
Arrangement of vibration devices on the seat. The upper and lower vibration devices present identical vibrations.

Four columns of vibration devices (model 602760, Foster Electric Company, Limited, Tokyo, Japan) were positioned at 80 mm intervals. The distance between the vibrating devices was determined based on the two-point discrimination threshold for the back, which is 40 mm [31]. This threshold represents the minimum distance at which two stimuli are perceived as distinct when applied to the skin. Vibration devices should be placed at distances exceeding this threshold. To effectively convey left–right spacing, we positioned them 80 mm apart, ensuring adequate coverage across the backrest of the wheelchair. To address instances where contact between the vibrating devices and the back was insufficient for some passengers, four rows of vibrating devices were installed in two layers. The upper and lower layers consistently presented identical vibrations (Figure 9).

The vibration devices were controlled by a microcontroller (STMicroelectronics Nucleo-F446RE, Geneva, Switzerland), which was connected to a laptop PC mounted on the wheelchair for mobility control. Information was presented to the passenger’s back using the method described in Section 3, based on the estimated position of the wheelchair.

**Figure 10:**
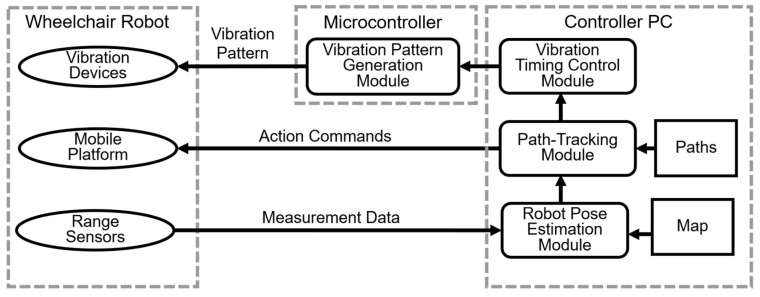
System architecture for wheelchair robot control and vibration feedback.

The system configuration is illustrated in Figure 10. The robot pose estimation module estimates the position and orientation of the wheelchair robot by processing measurement data from onboard range sensors and referencing a preloaded environmental map. Based on the estimated pose, the path-tracking module computes action commands to ensure the robot follows the designated path and controls the movement of the wheelchair. The vibration timing control module calculates the appropriate start time for vibration based on the proximity of the robot to the avoidance path. Subsequently, the vibration pattern generation module on the microcontroller generates the vibration pattern and transmits it to the vibration devices installed in the wheelchair seat, providing tactile feedback to the passenger.

### 4.4. Participants

Twenty healthy male adults (mean age: 23.3 years, standard deviation: 1.1) participated in our experiment. One participant was excluded from the evaluation due to an error in the movement path of the wheelchair during the experiment. All procedures in this study were approved by the Ethical Committee of Hiroshima City University. Written informed consent was obtained from all participants.

### 4.5. Evaluation

**Figure 11:**
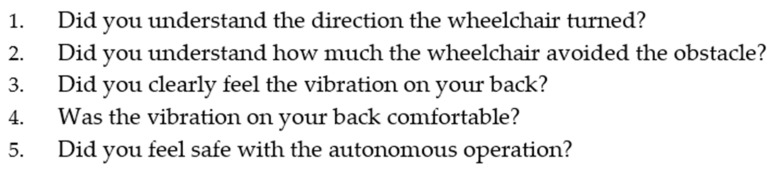
Questionnaire assessing user experience with autonomous wheelchair operation.

For each obstacle avoidance run performed by the wheelchair robot, participants rated five questions on a seven-point scale ranging from 1 (not applicable at all) to 7 (very applicable) using a questionnaire (Figure 11). In particular, we analyze the ratings for vibration clarity in Question 3 across all conditions, including Condition C, where no vibration was presented. This is because, when the wheelchair is used without vibration feedback, vibrations from movement itself may still be perceived in addition to those caused by the path presentation system. The perception of vibration clarity was evaluated to determine whether the path vibrations were sufficiently perceived and whether vibrations from the back of the wheelchair were distinguishable. Comparisons between conditions were analyzed using the Steel–Dwass nonparametric multiple comparison test, with a significance level of 1%.

### 4.6. Procedure

After receiving a written explanation of the purpose of the experiment, participants provided informed consent to participate. They were briefed on the experimental procedure, in which the autonomous wheelchair avoided obstacles and pedestrians in its path. Participants experienced all conditions listed in Table 1 to familiarize themselves with the wheelchair and the information presentation methods. Following this familiarization, each participant completed six experimental runs—two runs for each of the three conditions described in Table 1. The three conditions were administered in a randomized order for each participant to counterbalance potential order effects. In each condition, participants experienced two different avoidance widths: one large and one small. After each run, participants completed a questionnaire to evaluate the performance of the system. The direction of avoidance was predetermined so that half of the six runs involved rightward avoidance and the other half leftward avoidance. During the experiment, participants listened to white noise through headphones to eliminate external auditory distractions, such as the operational sounds of the vibrating devices.

## 5. Results

**Figure 12:**
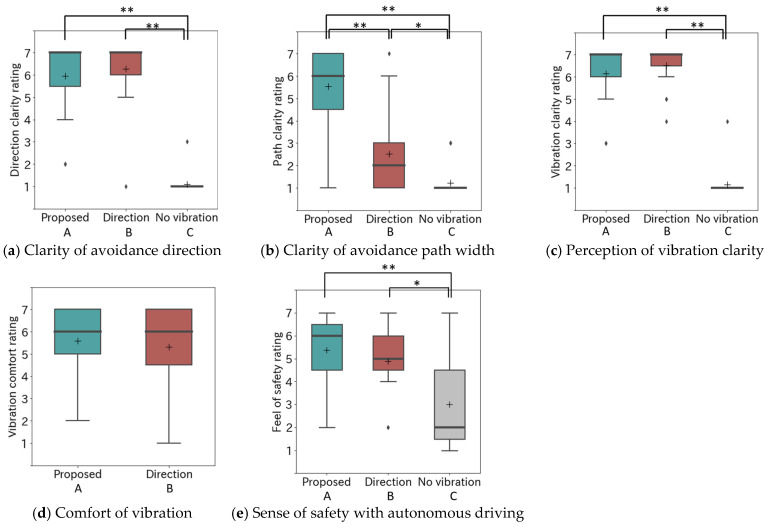
Results of the questionnaire evaluation (* indicates *p* < 0.05 and ** indicates *p* < 0.01).

Figure 12 shows the results of the post-experiment questionnaire following the driving experience. A complete list of participant evaluations is provided in Appendix A. For the clarity of the direction of avoidance (Figure 12a), both Conditions A and B received high ratings, with no significant difference between them (*p* = 0.86). Conditions A and B were rated significantly higher than Condition C, where no presentation was provided: A > C (*p* < 0.01) and B > C (*p* < 0.01).

Regarding the clarity of avoidance path width (Figure 12b), Condition A was rated significantly higher than Condition B, which presented only the direction of avoidance, and Condition C, which did not provide avoidance information: A > B (*p* < 0.01) and A > C (*p* < 0.01). Condition B tended to be higher than Condition C (*p* = 0.016), but the difference was not statistically significant.

For the sense of safety regarding the future mobility of the automated wheelchair (Figure 12e), Conditions A and B tended to be rated higher than Condition C, suggesting the effectiveness of using vibration to convey future travel paths. The median values for Conditions A and B were 6 and 5, respectively. Although Condition A was rated higher, the difference was not statistically significant (*p* = 0.438). Conditions A was rated significantly higher than Condition C (*p* < 0.01). Condition B tended to be higher than Condition C (*p* = 0.012), but the difference was not statistically significant.

The evaluation of vibration clarity and comfort was consistently high for both Conditions A and B. Regarding vibration clarity (Figure 12c), Conditions A and B were rated significantly higher than Condition C: A > C (*p* < 0.01), B > C (*p* < 0.01). In terms of vibration comfort, the median values for Conditions A and B were 6, indicating a sufficiently high rating. These results confirm that the presentation of information through vibration devices installed in the seat was effectively communicated to the passenger, resulting in no significant discomfort (Figure 12c,d).

## 6. Discussion

The primary contribution of this study is the proposal of a method for presenting future travel paths to passengers of an autonomous wheelchair using an array of tactile devices installed on the seat. The results demonstrated that this method was highly rated by passengers compared to conventional approaches. Previous research has focused on presenting navigation information during manual operation, showing the effectiveness of conveying details about future left–right turns and surrounding obstacles.

A key feature of the proposed method is its use of presentational motion, where information is conveyed through vibrotactile sensations that move smoothly. This approach likely contributed to the effective presentation of the degree of right/left turns. Participants commented on their sense of comfort, noting that “The pathway was clearer and felt more secure.”

The experimental results indicated that presenting not only right/left turns but also future paths to passengers of the autonomous wheelchair was effective. Several participants provided feedback, such as “Future pathways are more easily understood”, highlighting the efficacy of the proposed method. In the evaluation of how clearly the width of the avoidance path was communicated in Figure 12b, the proposed method A received significantly higher ratings than the conventional methods B and C, confirming its effectiveness in conveying the width of the future path. These results suggest that the proposed method clearly conveys both the direction and width of avoidance, along with the high rating for avoidance direction in Figure 12a, effectively conveying the future path of wheelchair movement. However, in Figure 12e, there was no significant difference in the perceived sense of security in autonomous driving between the proposed method A, which presented both the direction and width of the route, and method B, which presented only the direction, although method A showed a slight tendency toward higher ratings. Many participants expressed appreciation for the information presentation, with comments such as “I feel safer if I know the direction to turn” and “I feel safer because the vibration helps me understand the direction and timing of avoidance”. A possible explanation for this result is that participants noticed the contrast with Condition C, in which no information was presented, and thus paid less attention to the differences between Conditions A and B.

Our findings suggest that presenting the future path of the wheelchair via haptic feedback enhances users’ understanding of its movement and reduces uncertainty about its trajectory. This insight can be applied to interface design by integrating intuitive tactile cues corresponding to wheelchair movements, thereby improving user experience and safety. Furthermore, this study offers valuable insights for optimizing path presentation methods based on user preferences and cognitive load considerations.

In the proposed method, the values of α and β in Equation (1) influence the presentation of information to passengers. These parameters may be determined by two factors: the sensitivity of passengers to changes in avoidance width and the typical range of avoidance widths encountered in the operating environment of the wheelchair. To present a clearly perceptible change in s for a required change in avoidance width *w*, α should be set to a relatively large value. To determine the appropriate range of α for perceiving changes in avoidance width, it is crucial to establish the minimum perceivable *s*. Verifying the minimum perceivable *s* remains an important direction for future research. Conversely, as α increases, the information presentation time in Equation (2) also increases. For larger avoidance widths encountered in the environment, excessively long presentation times may compromise the effectiveness of warnings. Additionally, for smaller avoidance widths, such as when the wheelchair is avoiding pedestrians or obstacles, it is essential to ensure that *s* remains positive (*s* > 0). However, for very small values of *s*, the vibration device may activate at extremely short intervals, making the difference imperceptible. In this experiment, with α=0.3, an increase of 0.5 m in avoidance width resulted in an increase of 0.12 s in presentation time *s*, a change that is perceptible. Assuming that avoidance widths range from 0.5 m to 1.8 m in this environment, α=0.3 leads to a variation in T of 1.17 s. An offset of β=−0.15 was introduced to shorten the presentation time. However, this decision-making process has not been validated for other environments, and evaluating parameter selection for diverse settings remains an important area for future research.

The experiments in this study were conducted in a controlled laboratory environment. First, the proposed method may not effectively convey information in environments with many people or obstacles, particularly in scenarios involving continuous avoidance maneuvers or more complex paths. Building on the method used in this study, future work should explore approaches such as increasing the amount of information by arranging vibration devices in two dimensions, rather than limiting vibration transmission to left–right movements. In addition, road surface unevenness may make vibration-based information presentation more difficult for passengers to interpret. In such environments, vibration devices could also be placed not only on the back of the wheelchair but also on other locations, such as the seat surface. Furthermore, a promising approach would be to combine other modalities, such as visual presentation, to enhance information transmission reliability rather than relying solely on vibration.

In the experiments conducted in this study, the wheelchair maintained a constant speed. However, unpredictable changes in speed and acceleration occur when the wheelchair is actually being driven. Communicating this to passengers is another important future challenge for information presentation in autonomous wheelchairs.

This study did not evaluate the impact of clothing on vibration transmission. Since the experiment was conducted indoors, cases where passengers wore a heavy coat were not considered. However, vibrations are expected to be less effectively transmitted compared to when a shirt is worn. One possible approach is to install additional vibration devices on the wheelchair seat to mitigate this effect. Investigating the impact of clothing on vibration transmission remains an important area for future research.

This study has not been validated with a sufficiently large and diverse participant pool in terms of age, gender, and physical condition. As a result, this may introduce bias in the evaluation of vibration feedback.

## 7. Conclusions

To share future travel plans with passengers of an autonomous wheelchair, we proposed a method for presenting detailed future travel paths using a vibration device installed on the seat. The proposed method was evaluated in a scenario where the wheelchair autonomously avoided pedestrians and obstacles in a controlled structured environment by able-body users. The results demonstrated that the proposed method provided better path understanding and a higher sense of security for passengers compared to conventional methods. For autonomous wheelchairs, it is essential to present a precise future path to mitigate passenger anxiety. In this study, “path” refers to the geometric trajectory of the position of the wheelchair, independent of time. Providing passengers with information about this future path helps them anticipate the movement of the wheelchair and feel more secure. To address this issue, we developed a path presentation method using an array of vibration actuators embedded in the seat. The method conveys the degree of right or left turning and deceleration through sequential vibrations on the back of the seat. When introducing autonomous driving technology to wheelchairs, sharing information with passengers is crucial. Future work will focus on validating this method in real-world environments and developing more effective interfaces.

## Figures and Tables

**Table 1:** Experimental conditions.

Condition	Information Presentation
A	The future path was presented in advance (proposed method)
B	The direction of left or right avoidance was presented in advance
C	No path information

## Data Availability

The original contributions presented in the study are included in the article; further inquiries can be directed to the corresponding author.

## Appendix A. Participant Ratings for Each Condition

This appendix presents the five items shown in Figure 11, which were rated on a seven-point scale by 20 subjects under the three experimental conditions A, B, and C.

(a) Clarity of avoidance direction

Subject	1	2	3	4	5	6	7	8	9	10	11	12	13	14	15	16	17	18	19	20
A	7	7	7	5	7	7	7	7	5	6	6	7	7	7	7	2	4	7	6	2
B	7	7	7	7	7	6	7	7	5	6	7	7	1	7	6	6	7	7	5	7
C	1	1	1	1	1	1	1	1	1	1	1	3	1	1	1	1	1	1	1	1

(b) Clarity of avoidance path width

Subject	1	2	3	4	5	6	7	8	9	10	11	12	13	14	15	16	17	18	19	20
A	7	7	7	7	2	6	7	7	4	2	4	6	7	7	7	6	7	6	5	1
B	3	5	7	1	2	3	1	2	3	1	1	5	1	1	1	1	1	1	3	6
C	1	1	1	1	1	1	1	1	1	1	1	3	1	1	1	1	3	1	1	1

(c) Perception of vibration clarity

Subject	1	2	3	4	5	6	7	8	9	10	11	12	13	14	15	16	17	18	19	20
A	6	7	7	7	7	7	7	7	5	6	5	7	7	7	7	3	3	7	6	6
B	7	7	7	5	7	7	7	7	4	6	7	7	7	6	7	7	7	7	5	7
C	1	1	1	1	1	1	1	1	1	1	1	4	1	1	1	1	1	1	1	1

(d) Comfort of vibration

Subject	1	2	3	4	5	6	7	8	9	10	11	12	13	14	15	16	17	18	19	20
A	7	7	7	3	7	5	5	7	5	5	6	7	6	6	6	5	5	4	6	2
B	7	7	7	1	7	3	5	6	5	3	6	7	3	5	6	7	5	4	5	7

(e) Sense of safety with autonomous driving

Subject	1	2	3	4	5	6	7	8	9	10	11	12	13	14	15	16	17	18	19	20
A	5	6	7	7	6	3	6	7	6	3	6	6	6	6	7	5	7	4	3	2
B	5	5	7	5	7	2	5	5	6	2	5	7	4	6	5	5	5	2	4	6
C	2	1	7	1	6	2	2	1	4	1	2	5	5	3	2	4	6	1	2	2
